# Fabrication of Next-Generation Skin Scaffolds: Integrating Human Dermal Extracellular Matrix and Microbiota-Derived Postbiotics via 3D Bioprinting

**DOI:** 10.3390/polym17192647

**Published:** 2025-09-30

**Authors:** Sultan Golpek Aymelek, Billur Sezgin, Ahmet Ceylan, Fadime Kiran

**Affiliations:** 1 Pharmabiotic Technologies Research Laboratory, Department of Biology, Faculty of Science, Ankara University, 06100 Ankara, Türkiye; 2 Graduate School of Natural and Applied Sciences, Ankara University, 06110 Ankara, Türkiye; 3 Department of Plastic, Reconstructive and Aesthetic Surgery, Koc University School of Medicine, 34450 Istanbul, Türkiye; 4 Department of Histology and Embryology, Faculty of Veterinary Medicine, Ankara University, 06110 Ankara, Türkiye

**Keywords:** chronic wound, skin tissue engineering, 3D bioprinting, extracellular matrix, postbiotics

## Abstract

This study presents the development of an advanced three-dimensional (3D) bioprinted skin scaffold integrating sodium alginate (SA), gelatin (Gel), human skin-derived decellularized extracellular matrix (dECM), and microbiota-derived postbiotics. To ensure a biocompatible and functional ECM source, human skin samples collected during elective aesthetic surgical procedures were utilized. Following enzymatic treatment, the dermal layer was carefully separated from the epidermis and subjected to four different decellularization protocols. Among them, Protocol IV emerged as the most suitable, achieving significant DNA removal while maintaining the structural and biochemical integrity of the ECM, as confirmed by Fourier-transform infrared spectroscopy. Building on this optimized dECM-4, microbiota-derived postbiotics from *Limosilactobacillus reuteri* EIR/Spx-2 were incorporated to further enhance the scaffold’s bioactivity. Hybrid scaffolds were then fabricated using 7% Gel, 2% SA, 1% dECM-4, and 40 mg/mL postbiotics in five-layered grid structures via 3D bioprinting technology. Although this composition resulted in reduced mechanical strength, it exhibited improved hydrophilicity and biodegradability. Moreover, antimicrobial assays demonstrated inhibition zones of 16 mm and 13 mm against methicillin-resistant *Staphylococcus aureus* (MRSA, ATCC 43300) and *Pseudomonas aeruginosa* (ATCC 27853), respectively. Importantly, biocompatibility was confirmed through in vitro studies using human keratinocyte (HaCaT) cells, which adhered, proliferated, and maintained normal morphology over a 7-day culture period. Taken together, these findings suggest that the engineered hybrid scaffold provides both regenerative support and antimicrobial protection, making it a strong candidate for clinical applications, particularly in the management of chronic wounds.

## 1. Introduction

The skin, as the largest organ of the human body, constitutes a critical barrier directly interfacing with the external environment. Owing to this fundamental role, the skin functions as a primary defense mechanism against a multitude of threats, including physical trauma, microbial invasion, and chemical agents [1,2]. Nonetheless, this protective function simultaneously subjects the skin to an elevated risk of injury, which is conventionally categorized into two principal types as acute and chronic wounds. Acute wounds typically arise from traumatic events such as burns or lacerations and generally undergo healing within an 8- to 12-week period through a tightly regulated biological process [3]. Conversely, chronic wounds are characterized by a failure to progress through the normal phases of healing within an expected timeframe, often persisting for months or longer. These wounds commonly manifest in individuals with underlying comorbidities and represent a growing public health concern, frequently described as a silent pandemic due to their high prevalence and significant burden [4]. For instance, approximately 2.5% of the United States population experience a diminished quality of life attributable to chronic wounds. Moreover, the global demographic shift towards an aging population, coupled with the rising incidence of chronic diseases such as diabetes, obesity, and infection, continues to intensify the clinical, social, and economic impact of chronic wounds [5]. This escalating burden exerts considerable pressure on healthcare infrastructures worldwide while adversely affecting patient outcomes. Accordingly, the development of effective strategies for the management and treatment of chronic wounds remains a fundamental priority within both clinical and public health domains.

Crucially, effective wound healing fundamentally depends on the precisely coordinated proliferation of key cell types, particularly keratinocytes and fibroblasts, which are indispensable for both re-epithelialization and extracellular matrix remodeling [6]. Indeed, this cellular proliferation not only drives tissue regeneration but also restores the skin’s barrier function, thereby reestablishing its critical protective role. Importantly, the extracellular matrix (ECM), which occupies the interstitial spaces of native tissues, provides a crucial biochemical and biophysical environment that sustains cellular viability and function. Building directly upon this foundational role, decellularized ECM (dECM), produced by meticulously removing cellular components while preserving the native architecture and bioactive molecules, serves as an exemplary substrate for pivotal cellular processes including adhesion, migration, proliferation, and immune modulation—each of which is integral to successful wound repair [7]. Moreover, the complex and heterogeneous composition of dECM, which comprises multiple collagen types (I, III, IV, and VII), proteoglycans, glycosaminoglycans (GAGs), fibronectin, elastin, laminin, and diverse growth factors, creates a highly bioactive microenvironment that finely regulates cellular behavior. This sophisticated milieu not only promotes keratinocyte and fibroblast proliferation but also orchestrates the intricate processes of re-epithelialization and matrix remodeling that are vital for functional skin regeneration [7]. Therefore, recognizing the pivotal role of dECM, recent advances in tissue engineering have increasingly focused on the development of three-dimensional (3D) scaffolds that faithfully replicate the native tissue microenvironment by utilizing dECM, thus fostering optimal conditions for cell proliferation and subsequent tissue regeneration [6].

Despite considerable advancements in tissue engineering, the effective treatment of chronic wounds complicated by infection continues to represent a significant clinical challenge. This is primarily due to the complex nature of chronic wounds, which are often colonized by pathogenic bacteria capable of forming resilient biofilms that hinder healing and exacerbate inflammation [8,9]. Consequently, the integration of antimicrobial functionalities within wound-healing scaffolds has become an indispensable strategy to actively prevent microbial colonization and disrupt biofilm formation. In this context, there has been a growing focus on novel biotic-based therapies that deliver targeted antimicrobial effects while simultaneously preserving scaffold biocompatibility and promoting tissue regeneration. In particular, topical biotic therapies have gained substantial attention as a means to counteract the increasing incidence of wound infections. The International Scientific Association for Probiotics and Prebiotics (ISAPP) defines “postbiotics” as “a preparation of inanimate microorganisms and/or their components that confers a health benefit on the host” [10]. These preparations encompass a diverse array of bioactive components, including metabolites, cell fractions, lysates, short-chain fatty acids (SCFAs), extracellular polysaccharides (EPS), teichoic acids, bacteriocins, proteins, peptidoglycan-derived muropeptides, and pili-like structures. Notably, bacteriocins, EPS, and lipopeptides demonstrate potent antibacterial properties by inhibiting the biofilm formation of pathogenic bacteria prevalent in chronic wounds, thereby potentially reducing the emergence of antibiotic-resistant strains [11]. Moreover, given that postbiotics do not contain live microorganisms, they present a safer and more stable alternative to probiotics, minimizing the risk of adverse reactions while maintaining therapeutic efficacy [12].

Specifically, this study focuses on the integration of postbiotics into skin tissue engineering. The postbiotics employed are derived from *Limosilactobacillus reuteri* EIR/Spx-2 (NCBI accession number: PP951925), a bacterial strain isolated from the gut microbiota of long-lived organisms, as identified in our previous research [13]. These postbiotics have demonstrated antimicrobial activity against key skin pathogens and have been shown to significantly enhance cellular proliferation and migration, as well as antioxidant and anti-inflammatory responses in vitro, thereby highlighting their promising role in tissue regeneration. In light of these findings, the postbiotics were selected for incorporation into the scaffold with the expectation that the beneficial effects observed in vitro will be effectively translated in the 3D construct, ultimately promoting more efficient wound healing. Consequently, this research aims to address existing challenges in tissue engineering through the fabrication of a novel hybrid scaffold integrating human dermal extracellular matrix and microbiota-derived postbiotics that not only supports robust skin regeneration but also provides potent antimicrobial functionality. Thus, this approach represents a pioneering effort to combine advanced biomaterials with microbiota-derived postbiotics, paving the way for next-generation skin substitutes with enhanced therapeutic potential for chronic wound management.

## 2. Materials and Methods

### 2.1. Ethical Statement

**Figure 1:**
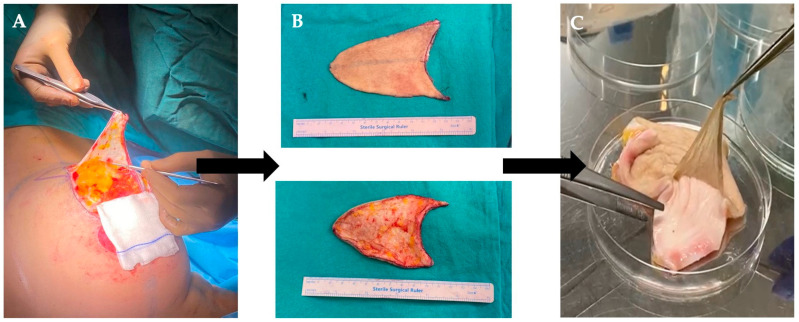
(**A**) Schematic representation of human skin tissue collection during surgical procedures. (**B**) Representative images of the excised full-thickness human skin tissue showing both the epidermal (outer) and dermal (inner) surfaces. (**C**) Manual separation of the epidermis from the underlying dermis using fine forceps following overnight enzymatic treatment.

Human skin tissue samples, used as a source of dECM preparation, were obtained from the Department of Plastic, Reconstructive and Aesthetic Surgery at Koc University School of Medicine, following approval by the Institutional Ethics Committee (protocol code: 2021.258.IRB2.051 and date of approval 6 May 2021). The skin samples were collected during clinically indicated surgical procedures from adult volunteers (aged 18 years or older) who had provided written informed consent to participate in the study (Figure 1A). The excised skin tissues (Figure 1B) which would otherwise be classified as medical waste, were ethically and legally repurposed for research in full compliance with institutional policies and regulatory standards.

### 2.2. Extraction of Human Skin-Derived dECM

Prior to decellularization process, a portion of each skin sample was reserved as a native tissue control and subsequently stored at −80 °C until further analysis. Initially, the skin samples were rinsed with sterile 0.9% sodium chloride solution (NaCl, Sigma-Aldrich, St. Louis, MO, USA) to eliminate any residual blood. Subsequently, subcutaneous fat tissue was carefully removed from the hypodermis layer. The tissue was then incubated overnight at 4 °C in a Dispase II enzyme solution (50 U/mL, Thermo Fisher, Waltham, MA, USA) to facilitate the enzymatic separation of the epidermis from the underlying dermis, which was subsequently performed using fine forceps (Figure 1C). Finally, the dermal tissue was carefully sectioned into 1 × 1 cm^2^ pieces and then subjected to four different decellularization protocols (Table 1). Importantly, all decellularization protocols were performed at room temperature with continuous agitation at 300 rpm.

Protocol I: Initially, the skin tissue samples were washed twice with 1× phosphate-buffer saline (PBS) selected for its isotonic and buffered properties that help maintain pH and osmotic balance, thereby supporting the structural integrity of the ECM during initial cleansing. Subsequently, the tissues were incubated in a 0.25% trypsin (Biological Industries, Beit Haemek, Israel) for 6 h to facilitate cellular detachment. The extended enzymatic incubation time was selected based on the action mechanism of trypsin, which requires several hours to effectively disrupt cell–matrix interactions while minimizing ECM damage. After this enzymatic treatment, the samples were rinsed three times with deionized water (dH_2_O). dH_2_O was used here as a hypotonic solution to assist in the lysis of any remaining cells and to flush out loosely bound cellular debris. The tissues were then immersed in 70% ethanol (Merck, Darmstadt, Germany) for 10 h, followed by a 15 min exposure to 3% hydrogen peroxide (H_2_O_2_, Merck, Germany) to achieve oxidative sterilization. Afterward, the samples underwent three additional washes with dH_2_O before being incubated for 22 h in a detergent solution containing 1% Triton X-100 (Sigma-Aldrich, St. Louis, MO, USA), 0.26% ethylenediamine tetraacetic acid (EDTA, Bioshop, Burlington, ON, Canada) and 0.69% tris buffer (Bioshop, Canada). Triton X-100, a non-ionic detergent, was chosen for its ability to disrupt lipid membranes while preserving protein–protein interactions, minimizing ECM damage. The detergent solution was refreshed after the first 6 h to maintain its effectiveness. Following detergent treatment, the tissues were rinsed with three times with dH_2_O and incubated for 2 h in a decontamination solution consisting of 0.1% peracetic acid (PAA, Sigma-Aldrich, St. Louis, MO, USA) and 4% ethanol, ensuring thorough microbial inactivation. Finally, the tissue samples were washed twice with 1× PBS and then with dH_2_O. The resulting dECM-1 was stored at −20 °C until further use [14].

Protocol II: Skin tissue samples were initially washed three times with dH_2_O to eliminate surface contaminants. The tissues were then incubated in 0.25% trypsin-EDTA (Sartorius, Göttingen, Germany) for 6 h to facilitate the removal of cellular components. Subsequently, the tissues were immersed in a solution 0.1% sodium dodecyl sulfate (SDS, Sigma-Aldrich, St. Louis, MO, USA) and 70% isopropanol (Merck, Germany) for 6 h. SDS, an ionic detergent, was used for its high decellularization efficiency through protein solubilization and membrane disruption. However, due to its harsh nature, extensive washing steps including five consecutive washes with dH_2_O were implemented to minimize ECM damage and prevent cytotoxicity in downstream applications. This was followed by a 12 h incubation in a solution consisting of 1% triton X-100 and 70% isopropanol, with the solution replaced every 4 h to preserve its effectiveness. After this step, the tissues were washed an additional five times with dH_2_O to eliminate residual detergent and alcohol. Finally, the samples were incubated in 100% isopropanol for 12 h, followed by six washes with dH_2_O to thoroughly remove any remaining chemicals. Upon completion of these steps, the dECM-2 was stored at −20 °C until further use [15].

Protocol III: Initially, skin tissue samples were washed three times with dH_2_O, which acts as a hypotonic solution to promote osmotic imbalance and facilitate early cell lysis, while also aiding in the removal of loosely bound impurities. The tissues were then incubated for 12 h in a hypotonic solution containing 10 mM tris-hydrochloride (Tris HCl, Serva, Heidelberg, Germany) 5 mM EDTA, and 1 μM phenylmethylsulfanyl fluoride (PMSF, Thermo Fisher, USA) to promote cell lysis. This was followed by a 24 h incubation in a hypertonic solution with 0.5% Triton X-100. The detergent solution was refreshed every 6 h to maintain efficacy. Afterward, the tissues were washed for 2 h with 1× PBS before undergoing treatment with 0.7% SDS solution for 24 h, with the detergent solution being replaced every 6 h to maintain consistent activity. SDS, an ionic detergent, was included to more aggressively solubilize cellular and nuclear proteins, enhancing decellularization efficiency. However, its use was carefully timed and followed by multiple PBS washes to minimize potential damage to ECM integrity. The samples were then immersed in a decontamination solution comprising 0.05% PAA and 3% ethanol for 3 h to ensure microbial inactivation. Finally, following thorough washing with 1× PBS to remove any residual chemicals, the dECM-3 was stored at −20 °C until further use [16].

Protocol IV: Similarly, Protocol IV employed a sequential hypotonic–hypertonic strategy. However, this protocol involved two cycles of alternating treatments to enhance cellular disruption. The initial wash with 1× PBS for 1 h helped stabilize the tissue environment through isotonic conditions. Following this step, the tissues were incubated for 8 h in a hypotonic solution consisting of 10 mM tris-HCl, 5 mM EDTA, and 1 μM PMSF to induce cell swelling and lysis. The samples were then transferred to a hypertonic solution containing 50 mM tris-HCl, 1 M NaCl, 10 mM EDTA, and 1 μM PMSF, and incubated for an additional 8 h. The combination of high salt concentration and buffering agents contributed to further cell rupture and dissociation of nuclear material. This hypotonic–hypertonic cycle was repeated once more to increase decellularization efficiency without relying on harsh detergents like SDS. Final PBS washes were extended to three cycles of 2 h each to ensure complete removal of residual reagents. As in other protocols, the resulting dECM-4 was stored at −20 °C until further use [17].

### 2.3. Characterization of dECM

To assess the efficiency of the decellularization process, native human skin tissue that had not undergone any decellularization was included as a positive control in all characterization analyses, enabling a direct comparison between untreated and processed samples.

#### 2.3.1. Quantification of DNA Content

To evaluate the efficiency of the decellularization process in removing cellular material, the DNA content of both native human skin tissue and dECM samples was quantified. Genomic DNA was extracted using the FavorPrep™ Tissue Genomic DNA Extraction Kit (Favorgen Biotech Corp., Ping-Tung, Taiwan), following the manufacturer’s instructions. The concentration and purity of the extracted DNA were assessed using an Epoch Microplate Reader (BioTek Instruments, Winooski, VT, USA) by measuring absorbance at 260/280 nm, which provides an indication of nucleic acid content and potential protein contamination.

#### 2.3.2. Histological Evaluation and Immunofluorescence Analysis

To evaluate the morphological characteristics of both dECM and native human skin tissue, samples were first embedded in paraffin blocks and then sectioned into thin slices of 5 μm thickness. General histological features were assessed using Mallory’s trichrome staining protocol, as modified by Crossmon [18], which effectively highlights connective tissue structures. To specifically identify GAGs within the samples, staining was performed with Alcian Blue (AB, Sigma-Aldrich, St. Louis, MO, USA) at two different pH levels (1.0 and 2.5), as well as Toluidine Blue (TB, Sigma-Aldrich, St. Louis, MO, USA), which are widely used for detecting acidic polysaccharides. Furthermore, a commercial trichrome staining kit (Invitrogen, Carlsbad, CA, USA) was utilized to visualize collagenous fibers in the connective tissue with enhanced specificity. All stained sections were carefully examined, and high-resolution images were captured using a light microscope (Olympus, Tokyo, Japan) to document the tissue architecture and dECM composition.

For detailed immunofluorescence analysis, the dECM and native tissue samples underwent fixation in 10% buffered neutral formalin (Sigma-Aldrich, St. Louis, MO, USA) for 18 h. Subsequently, samples were embedded in paraffin and sectioned into 4 μm slices. To prevent non-specific binding of antibodies, sections were incubated at room temperature with 10% normal goat serum (Thermo Fisher, USA) for 10 min, effectively blocking potential background staining. The primary antibody targeting Collagen Type I Alpha 1 Chain (anti-Col1α1, Abcam, Cambridge, UK) was then applied, and sections were incubated at 4 °C for 12 h to allow for optimal antigen–antibody binding. Following this, the sections were treated with a fluorescein isothiocyanate (FITC)-conjugated goat anti-rabbit secondary antibody (Abcam, UK) in a humidified chamber at 37 °C for 30 min, facilitating fluorescent labeling of the targeted collagen. Prior to each antibody incubation, the sections were thoroughly washed with 1× PBS for 10 min to remove any unbound antibodies and reduce background fluorescence. After completion of the immunostaining protocol, nuclei were counterstained with 4′,6-diamidino-2-phenylindole (DAPI, Sigma-Aldrich, USA), providing clear visualization of cell nuclei. Finally, all samples were visualized using a fluorescence microscope (Olympus, Japan) equipped with a digital camera system, enabling high-quality imaging for qualitative and quantitative analysis of collagen distribution within the dECM.

#### 2.3.3. Fourier Transform Infrared Spectroscopy

Fourier Transform Infrared Spectroscopy (FTIR) analysis was conducted to investigate the chemical bond similarities between the dECM and native tissue samples, as well as to identify the functional groups present within both structures. The analysis was performed using the attenuated total reflectance (ATR) technique on an IR Affinity-1 Infrared Spectrometer (Shimadzu, Kyoto, Japan). Spectral data were collected over a range of 500 to 4000 cm^−1^ with a spectral resolution of 4 cm^−1^. To ensure accuracy, the background absorbance was recorded with 100 scans prior to sample measurement.

### 2.4. Extraction of the Microbiota-Derived Postbiotics

The bacterial strain *Limosilactobacillus reuteri* EIR/Spx-2, obtained from the Pharmabiotic Technologies Research Laboratory culture collection, was used as the source for postbiotics production [13]. Glycerol-preserved bacterial stock stored at −80 °C were inoculated at 1% (*v*/*v*) into 10 mL of De Man, Rogosa, and Sharpe (MRS, Merck, Germany) broth and incubated aerobically at 37 °C for 24 h. After two consecutive activation steps to ensure culture viability and purity confirmed by streak plating on solid medium, the active culture was further inoculated at 1% into 200 mL MRS broth and incubated at 37 °C for 24 h. Following the incubation period, the culture was centrifuged at 15,000× *g* for 20 min to separate the postbiotics-containing supernatant. To ensure complete removal of bacterial cells, the supernatant was filtered through sterile 0.22 µm membrane filters (Sartorius, Germany). The cell-free supernatant was subsequently frozen sequentially at −20 °C and −80 °C, then lyophilized under vacuum (0.120 mBar) with a condenser temperature of −58 °C (Buchi, Flawil, Switzerland). The resulting powder, including postbiotics, was stored at −20 °C until further use [19].

### 2.5. Fabrication of Hybrid Scaffolds

#### 2.5.1. Preparation of dECM Based Hydrogel

dECM (10 mg) was enzymatically digested in 1 mg/mL pepsin solution prepared in 0.01 N HCl under constant magnetic stirring at room temperature for 72 h. During this process, pepsin selectively cleaves the telopeptide regions of collagen, solubilizing the ECM into a pre-gel solution composed primarily of monomeric collagen and other ECM proteins. This enzymatic digestion step facilitates the formation of a homogeneous mixture by disrupting fibrillar aggregates. The digest was then neutralized by titration with 1 M NaOH at a volumetric ratio of 1:10, followed by adjustment of ionic strength and pH through the addition of 10× PBS at a 1:9 ratio. Upon neutralization, collagen and other ECM proteins spontaneously reorganize into a three-dimensional network through an entropy-driven mechanism [20,21,22]. Gelation was carried out at 37 °C for 60 min, yielding a stable hydrogel. The initial assessment of gel stability was performed using a macroscopic tube inversion test, wherein the absence of flow indicated successful gel formation [20]. The resultant hydrogel was stored at 4 °C prior to utilization in 3D bioprinting protocols.

#### 2.5.2. Preparation of Hybrid Bio-Ink

Hybrid bio-inks were prepared by mixing sodium alginate (SA), gelatin (Gel), dECM based hydrogel, and postbiotics in specific concentrations to achieve the desired biological and printing properties. Group 1 (G1) contained 2% (*w*/*v*) SA and 7% (*w*/*v*) Gel, serving as the basic formulation without dECM or postbiotics. Group 2 (G2) included 1% (*w*/*v*) dECM hydrogel added to G1 to improve the biological composition and mimic native extracellular matrix features. Group 3 (G3) incorporated 40 mg/mL postbiotics together with 1% dECM hydrogel, 2% SA, and 7% Gel. These concentrations were selected based on a combination of preliminary experiments, which demonstrated that 2% SA provides adequate viscosity and gelation properties for bioprinting, while 7% Gel supports cell adhesion and structural stability without compromising printability or mechanical integrity. The concentration of postbiotics in G3 was chosen based on the minimum inhibitory concentration (MIC) determined in our previous studies, which showed effective antimicrobial activity against important skin pathogens [13]. All components were stirred continuously at 37 °C for 1 h to achieve a uniform and stable mixture suitable for 3D bioprinting. After incubation, the bio-inks exhibited consistent viscosity and homogeneity used for scaffold fabrication

#### 2.5.3. Three-Dimensional Bioprinting

The prepared hybrid bio-ink for each group was aseptically transferred into a sterile polyethylene cartridge compatible with a low-temperature printhead (operating range 0–70 °C) designed specifically for 3D bioprinting applications. A 0.20 mm needle adaptor was securely attached to the cartridge before placing it into the cartridge holder of the 3D bioprinter (EnvisionTEC, Dearborn, MI, USA). To collect the printed constructs, a sterile 60 mm Petri dish with its lid removed was positioned at the center of the printing platform. Printing parameters were precisely set using the bioprinter’s control software as follows: pneumatic pressure at 1.8 bar, printing speed at 6.5 mm/s, and a total of five layers per construct. Upon completion of printing, the constructs were immediately subjected to ionic crosslinking by immersion in a 1 M calcium chloride (CaCl_2_, Sigma-Aldrich, St. Louis, MO, USA) solution for 15 min to stabilize their structure. Finally, the crosslinked scaffolds were carefully removed from the solution and stored at 4 °C until further use.

### 2.6. Chracterization of 3D Bioprinted Scaffold

#### 2.6.1. Mechanical Properties

The mechanical properties of the hybrid scaffolds were systematically evaluated using a universal tensile and compression testing machine (Shimadzu, Japan). To determine the key mechanical parameters including ultimate tensile strength, elongation at break, and Young’s modulus, tensile testing was evaluated. Briefly, each scaffold sample was firmly secured between the device grips and subjected to uniaxial tensile loading at a constant elongation rate of 1 mm/min until structural failure occurred, with a maximum force limit of 50 N applied. Compression tests were subsequently performed on the same instrument, applying a compressive load at a rate of 40 mm/s to simulate mechanical stress conditions relevant to physiological environments. Additionally, the thickness of each scaffold specimen was accurately measured using a digital micrometer prior to testing to standardize sample dimensions and enable precise calculation of stress and strain values.

#### 2.6.2. Swelling and Biodegradation Behavior

The swelling behavior of the hybrid scaffolds was quantitatively assessed to determine their capacity for water uptake, which is essential for maintaining a hydrated wound environment. Scaffold samples were weighed to obtain the initial mass (*M*_0_) before immersion in 1× PBS at 37 °C. Samples were withdrawn at specific time points (3, 12, 24, 48, and 72 h), and excess surface fluid was carefully removed using filter paper to avoid measurement artifacts. The swollen mass (*M*_t_) was recorded immediately thereafter. The swelling ratio was calculated as a percentage increase relative to the initial mass using the following equation [17]:

$$S\left(\%\right)=\left[\frac{\left(M_{t}-M_{0}\right)}{M_{0}}\right]\times \;100.$$

For the biodegradation assay, scaffolds were incubated in 3.2 U/mL collagenase type I solution (Sigma-Aldrich, St. Louis, MO, USA) at 37 °C to simulate enzymatic degradation conditions relevant to in vivo remodeling. The initial weight (*M*_0_) was recorded prior to incubation. Samples were retrieved on days 1, 3, 5, and 7, gently rinsed with dH_2_O, and surface moisture was blotted prior to reweighing to obtain the residual mass (*M*_t_). The percentage degradation was calculated as follows [17]:

$$D\left(\%\right)=\left[\frac{\left(M_{t}-M_{0}\right)}{M_{0}}\right]\times \;100.$$

#### 2.6.3. Antibacterial Properties

The antibacterial performance of the postbiotics-loaded hybrid scaffolds (G3) was quantitatively evaluated against methicillin-resistant *Staphylococcus aureus* (MRSA, ATCC 43300) and *Pseudomonas aeruginosa* (ATCC 27853) using the standard agar well diffusion method. The bacterial strains were first cultivated in Brain Heart Infusion (BHI, Merck, Germany) broth under aerobic conditions at 37 °C for 18–24 h. After incubation, the cultures were adjusted to a turbidity corresponding to the 0.5 McFarland standard (~1 × 10^8^ CFU/mL) using a densitometer to ensure a standardized inoculum. Following standardization, 10 mL of semi-solid BHI medium (0.75% agar), inoculated with each bacterial suspension, was poured onto BHI agar plates (1.5% agar) to form a uniform bacterial lawn. Once the overlay solidified, wells (5 mm diameter) were aseptically created in the agar, and G3 hydrogel samples, previously sterilized by ultraviolet (UV) irradiation for 30 min were carefully loaded into each well. The plates were then incubated at 37 °C for 24 h. Antibacterial activity was assessed by measuring the diameter of the inhibition zones (in mm) formed around each hydrogel sample using a precision digital caliper. Group 2 (G2) scaffolds, which lacked postbiotics content, were used as negative controls to verify that any observed antibacterial activity was attributable specifically to the postbiotics agent incorporated within the G3 formulation.

#### 2.6.4. Biocompatibility and Cytoskeletal Evaluation

To evaluate scaffold biocompatibility, human keratinocyte (HaCaT, ATCC HB-8930) cells were used. Cells were cultured in Dulbecco’s Modified Eagle Medium (DMEM; Sigma-Aldrich, St. Louis, MO, USA) supplemented with 1% L-glutamine (Gibco, UK), 1% penicillin/streptomycin (Gibco, UK), and 10% fetal bovine serum (FBS; Gibco, UK) under standard incubation conditions (37 °C, 5% CO_2_, 90% humidity), with medium renewal every other day until ~80% confluency was reached. At confluency, cells were detached using trypsin/EDTA (Sartorius, Germany) and collected by centrifugation at 200× *g* for 5 min. Viability was assessed by trypan blue (Sigma-Aldrich, St. Louis, MO, USA) staining and counted with an automated cell counter (Bio-Rad, Hercules, CA, USA). Scaffolds were sterilized under UV exposure for 30 min to ensure aseptic conditions and then positioned into wells of standard cell culture plates. Subsequently, HaCaT cells were seeded onto the scaffolds at a density of 1 × 10^5^ viable cells per construct. Cultures were maintained for 7 days under standard incubation conditions (37 °C, 5% CO_2_, and 90% humidity), with the culture medium being refreshed on Day 3 to sustain optimal nutrient availability. As a control group, the same number of cells was seeded onto sterile 12 mm glass coverslips, serving as a 2D substrate for comparative analysis of cell behavior. Cell viability on Days 1 and 7 post-seeding was visualized using a Live/Dead fluorescence staining assay (Thermo Fisher, USA), and imaging was performed using a fluorescence microscope (Olympus, Japan) equipped with a digital camera system.

To examine cytoskeletal morphology, actin filament staining was performed on Day 7. Following the viability assay, samples were rinsed twice with 1× PBS and fixed in 4% paraformaldehyde (Thermo Fisher, USA) for 15 min at room temperature. Fixative residues were removed with three PBS washes, followed by permeabilization in 0.1% Tween-20 (Sigma-Aldrich, St. Louis, MO, USA) for 15 min. After further washing, a blocking step was conducted with 4% bovine serum albumin (BSA; Sartorius, Germany) in 0.1% Tween-20 for 1 h to minimize nonspecific binding. Samples were then incubated overnight at 4 °C with primary anti-actin antibody (1:100, Thermo Fisher, USA), followed by two washes in 0.1% Tween-20 (each 3 min), and incubation with a secondary Goat anti-Mouse IgG antibody (1:200, Thermo Fisher, USA) for 1 h at room temperature. Following secondary antibody incubation, the samples were subjected to two additional 5 min washes in 0.1% Tween-20 to minimize background fluorescence. Subsequently, the sections were counterstained with DAPI (Thermo Fisher, USA) to visualize cell nuclei and mounted with coverslips. Final imaging was performed using a laser scanning confocal microscope (Carl Zeiss Microscopy GmbH, Jena, Germany).

### 2.7. Statistical Analysis

All experiments were conducted in two independent studies with three biological replicates each (*n* = 3). The data were analyzed using one-way ANOVA. Dunnett’s test was used to compare each group with the control, and Tukey’s test for pairwise comparisons. Analyses were performed using GraphPad Prism 10.2.1. Results are shown as mean ± SD, with *p* < 0.05 considered statistically significant.

## 3. Results

### 3.1. Selection of Ideal Decellularization Protocol and Its Characterization

**Figure 2:**
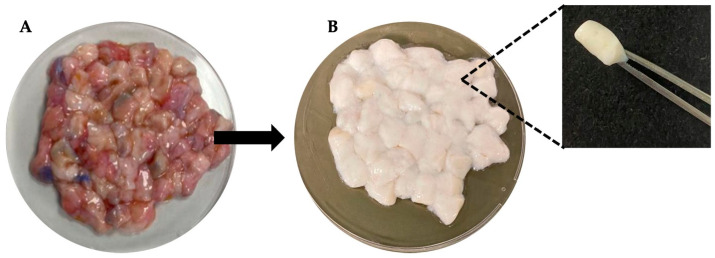
(**A**,**B**) Macroscopic appearance of human dermal tissue before and after decellularization.

The dermal tissues surgically obtained human skin samples were subjected to four distinct decellularization protocols. Prior to decellularization, native human dermal tissue exhibited a reddish appearance, primarily due to the presence of cellular components, including hemoglobin-rich erythrocytes and other nucleated cells (Figure 2A). Following the application of all decellularization protocols, a noticeable macroscopic color transition from red to white was observed, macroscopically (Figure 2B).

**Figure 3:**
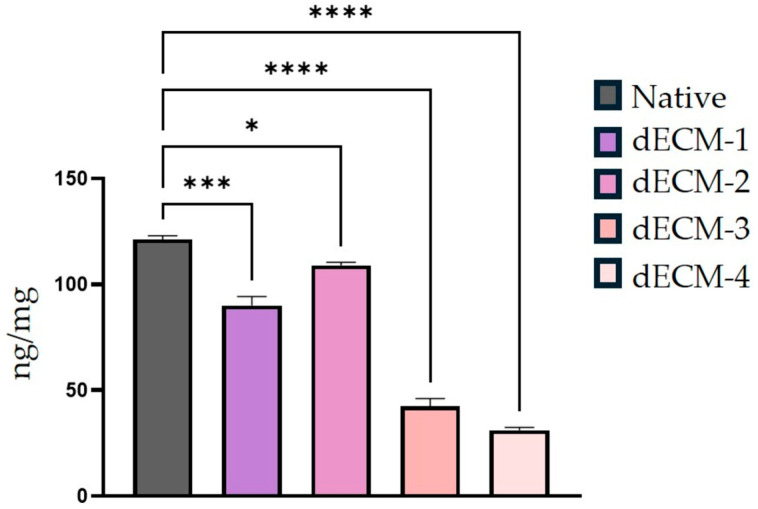
Quantitative analysis of residual DNA content in native and decellularized human dermal tissues following application of four distinct decellularization protocols. DNA concentrations are presented as mean ± standard deviation (SD). Statistical significance was determined by one-way ANOVA followed by Dunnett’s multiple comparison test (* *p* < 0.05, *** *p* < 0.001, **** *p* < 0.0001).

To evaluate the efficiency of the decellularization protocols, quantitative analysis of residual DNA content was conducted. According to our results, native human dermal tissue exhibited a baseline DNA concentration of 110 ± 15 ng/mg. Following treatment, dECM-1, dECM-3 and dECM-4, demonstrated statistically significant reductions in DNA content, measured as 80 ± 8 ng/mg (*p* < 0.001), 40 ± 6 ng/mg (*p* < 0.0001), and 30 ± 2 ng/mg (*p* < 0.0001), respectively. In contrast, dECM-2 resulted in a DNA concentration of 105 ± 5 ng/mg (*p* = 0.0191), which was not considered biologically significant when compared to the native tissue (Figure 3). Accordingly, the pronounced reduction in DNA content observed in dECM-3 and dECM-4, remaining below the widely accepted threshold of ≤50 ng/mg for effective decellularization, led to the selection of these protocols for subsequent detailed characterization [23].

**Figure 4:**
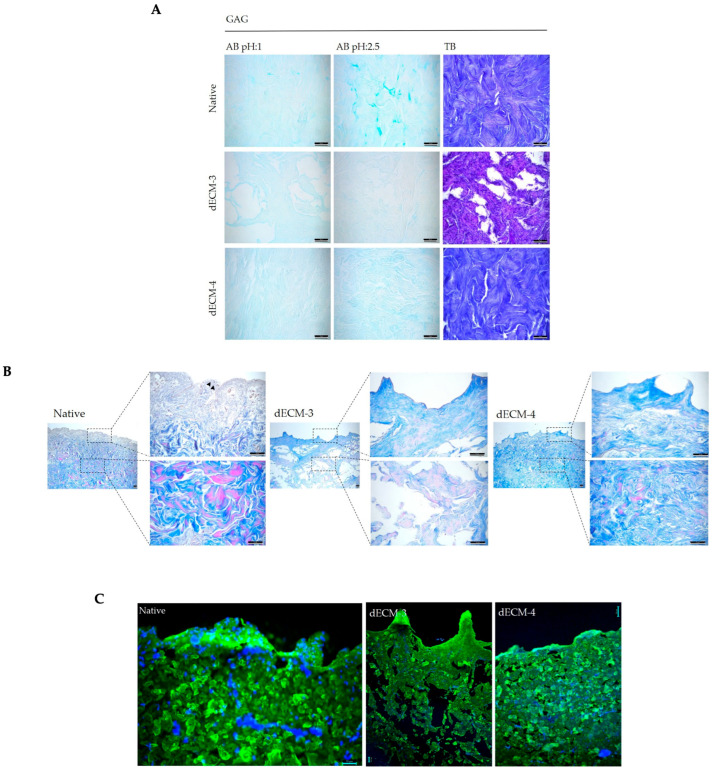
Histological and immunofluorescence evaluation of dECM-3 and dECM-4. (**A**) Glycosaminoglycan preservation assessed by Alcian Blue (AB) staining and Toluidine Blue (TB) staining (Scale bar = 50 µm). (**B**) Collagen fiber integrity evaluated by Masson’s Trichrome staining, which stains collagen fibers blue and cell nuclei brown (Scale bar = 200, 100, 50 µm). (**C**) Immunofluorescence analysis targeting collagen type I alpha 1 chain (Col1α1, green) and nuclei (DAPI, blue), arrows highlight regions exhibiting loss of fiber organization and structural integrity (Scale bar = 50 µm).

To further determine the most effective decellularized tissue between dECM-3 and dECM-4, previously identified based on DNA quantification, comprehensive histological analyses were conducted. The preservation of GAGs was evaluated using Alcian Blue (AB) staining at pH 1.0 and 2.5, as well as Toluidine Blue (TB) staining, both of which are well-established methods for detecting acidic polysaccharides in ECM components. dECM-4 exhibited a GAG profile closely resembling that of native tissue, whereas dECM-3 showed significant degradation and loss of GAG content (Figure 4A). Collagen fiber integrity was assessed by Masson’s Trichrome staining, which distinctly visualizes collagen fibers in blue and cell nuclei in brown. Absence of nuclei in decellularized samples confirmed effective removal of cellular material; however, collagen preservation was markedly superior in dECM-4 compared to dECM-3 (Figure 4B). These histological observations were corroborated by immunofluorescence analysis targeting collagen type I alpha 1 chain (Col1α1) and nuclear staining with DAPI. dECM-4 demonstrated enhanced retention of collagen architecture alongside minimal residual nuclear material, further validating its efficacy in maintaining ECM integrity post-decellularization (Figure 4C).

**Figure 5:**
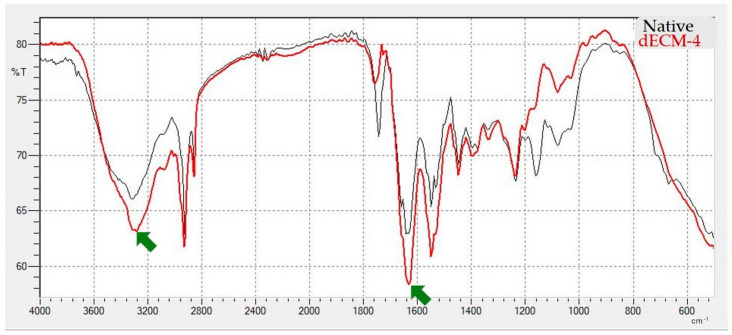
Fourier Transform Infrared spectra comparing native human dermal tissue and dECM-4. The arrows indicate characteristic absorption peaks at approximately 1632 cm^−1^ and 3300 cm^−1^.

Due to its superior performance in preserving ECM architecture, minimizing residual DNA content, and maintaining key biochemical components such as GAG and collagen, Protocol IV was selected as the most optimal decellularization protocol. To further validate the biochemical preservation of ECM components, FTIR was performed to assess the chemical similarity between native dermal tissue and the dECM-4. Both spectra exhibit characteristic absorption peaks indicative of preserved extracellular matrix composition. The prominent peak at ~1632 cm^−1^ corresponds to the C=O stretching vibration of amide I, while the broad band around ~3300 cm^−1^ is attributed to N–H stretching vibrations of amide A, both of which are representative of collagen-rich structures. The spectral similarity between native and decellularized samples suggests that Protocol IV effectively maintains the biochemical integrity of the ECM following decellularization (Figure 5).

### 3.2. Evaluation of Hybrid Scaffold Architecture and Functional Properties

**Figure 6:**
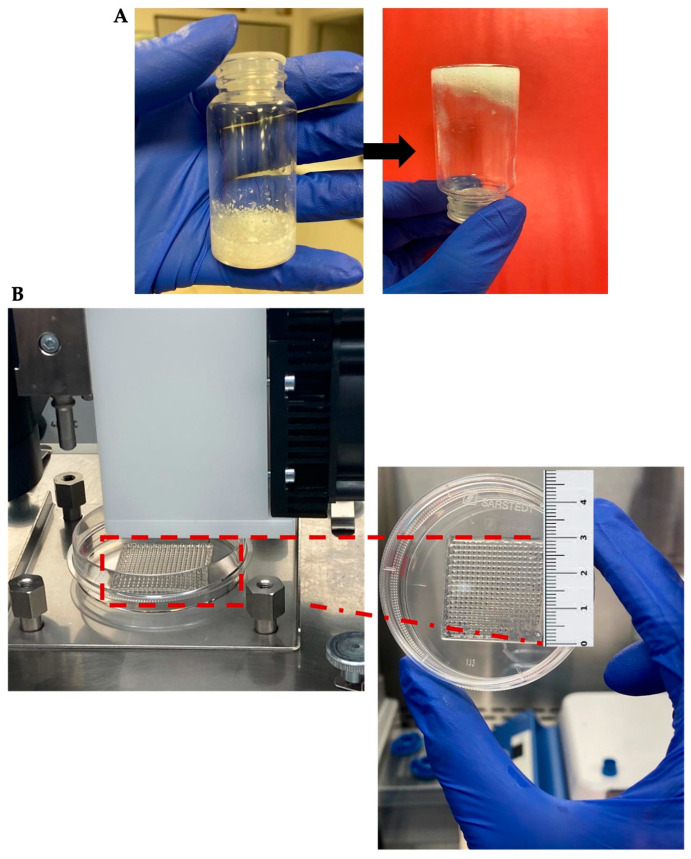
(**A**) Evaluation of hydrogel gelation and structural stability using the macroscopic tube inversion test. (**B**) Representative image of the five-layered, grid-patterned constructs (3 cm × 3 cm) fabricated via 3D bioprinting.

The bioactive hydrogel form of dECM-4 was first evaluated using a macroscopic tube inversion test, in which no flow was observed upon tube inversion (Figure 6A) thereby representing a critical advancement in the development of a biomimetic scaffold suitable for 3D bioprinting. Following this, Groups G1, G2, and G3 were subsequently subjected to a 3D bioprinting procedure using optimized printing parameters. As a result, five-layered, grid-patterned constructs with precise dimensions (3 cm × 3 cm) and a thickness of approximately 1.48 mm were successfully fabricated (Figure 6B).

**Figure 7:**
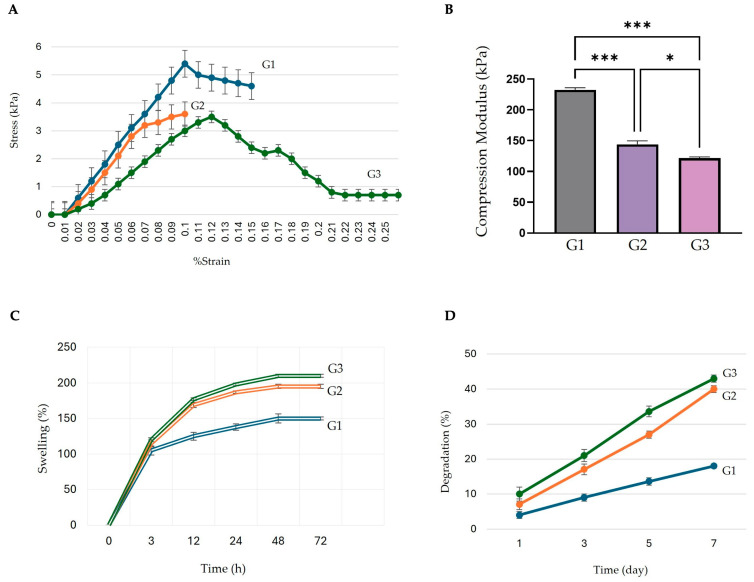
Mechanical and physicochemical characterization of hybrid scaffolds. (**A**) Tensile strength. (**B**) Compression modulus. (**C**) Swelling behavior over 72 h. (**D**) Degradation profile over 7 days for groups G1, G2, and G3. The data is presented as mean ± standard deviation (SD). Statistical significance was determined by one-way ANOVA followed by Dunnett’s multiple comparison test (* *p* < 0.05, *** *p* < 0.001).

Following the successful fabrication of the hybrid scaffolds, comprehensive characterization tests were performed to evaluate their mechanical properties, swelling behavior, and degradation profiles. Tensile testing showed that the SA/Gel control group (G1) exhibited the highest maximum stress of approximately 5.3 kPa at 10% strain, outperforming the groups dECM-4 (G2) and dECM-4 plus postbiotics (G3), which demonstrated maximum stress values of 3.7 kPa and 3.5 kPa, respectively (Figure 7A). Consistently, Young’s modulus demonstrated a decreasing trend in scaffold stiffness, with measurements of 55 kPa for G1, 38 kPa for G2, and 25 kPa for G3. The compressive modulus values were measured as 230 kPa for G1, 140 kPa for G2, and 90 kPa for G3 (Figure 7B). Swelling assays indicated rapid water absorption in all groups, with G3 exhibiting the highest swelling ratio (~210%) at 72 h (Figure 7C). Degradation profiles showed a time-dependent mass loss over 7 days, with the G3 undergoing the most extensive degradation (43%), followed by G2 (40%) and G1 (17%) (Figure 7D).

**Figure 8:**
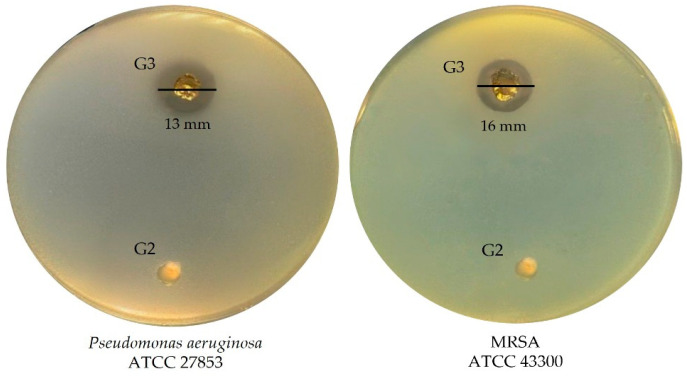
Antibacterial activity against important skin pathogens.

To assess the potential of the fabricated scaffolds in preventing bacterial infections, their antibacterial activity against *P. aeruginosa* ATCC 27853 and MRSA ATCC 43300 strains was evaluated using the well diffusion method. No antibacterial effect was observed in the scaffold lacking postbiotics (G2), whereas the scaffold incorporating postbiotics (G3) exhibited significant antibacterial activity. The inhibition zone diameters measured were 13 ± 1.8 mm against *P. aeruginosa* ATCC 27853 and 16 ± 1.2 mm against MRSA ATCC 43300 (Figure 8), demonstrating the efficacy of postbiotics incorporation in enhancing antimicrobial properties.

**Figure 9:**
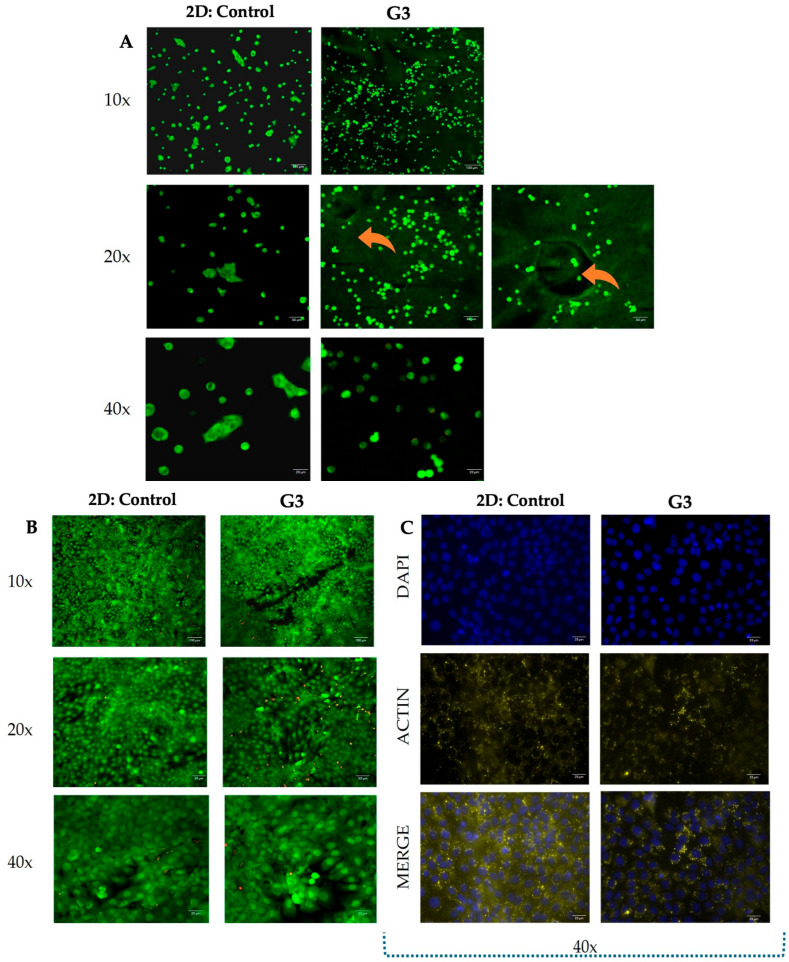
Biocompatibility assessment of the G3 scaffold. (**A**) Live/Dead staining on Day 1 showing high cell viability (green). (**B**) Cell morphology and colonization on Day 7. (**C**) Immunofluorescence of F-actin (yellow) and DAPI (blue) on Day 7 confirming cytoskeletal organization and uniform cell distribution. Arrows indicate scaffold grid structures.

The biocompatibility of the G3 scaffold was evaluated through Live/Dead fluorescence staining to assess cell viability and morphology over time. At Day 1 post-seeding, cells cultured on the G3 scaffold exhibited high viability, although they had not yet fully developed their typical morphology; notably, the viability rates were significantly higher compared to the control group (Figure 9A). By Day 7, cells displayed well-defined morphological features and extensive colonization throughout the scaffold, with most cells remaining viable in both the G3 and control groups despite a minor presence of dead cells (Figure 9B). Additionally, immunofluorescent staining of F-actin at Day 7 demonstrated an organized cytoskeletal architecture and confirmed effective cellular spreading, while DAPI staining revealed uniform nuclear distribution, indicating consistent cell attachment and proliferation across the scaffold (Figure 9C).

## 4. Discussion

Chronic wounds represent a significant public health concern, adversely impacting patients’ quality of life while imposing a substantial economic burden on healthcare systems. Due to their long healing times, higher risk of infection, reduced mobility, and overall decline in well-being, chronic wounds remain a serious clinical challenge. Conventional treatment modalities, including moist dressings, antibiotics, and debridement, frequently prove inadequate in effectively supporting the sequential phases of wound healing namely, hemostasis, inflammation, proliferation, and remodeling, and consequently fail to sufficiently promote tissue regeneration [24]. Moreover, in cases where chronic wounds do not heal or become infected, patients face severe complications such as sepsis or amputation, which markedly elevate mortality rates [25]. Therefore, tissue engineering strategies aimed at the development of biologically active wound dressings capable of accelerating the healing process have attracted considerable research interest.

In recent decades, skin tissue engineering has emerged as a pivotal field within regenerative medicine, driven by the increasing demand for effective skin substitutes to address burns, chronic wounds, and various dermatological conditions. Accordingly, the global regenerative medicine market is rapidly expanding, valued at USD 29.42 billion in 2023 and projected to reach USD 154.05 billion by 2033, with a compound annual growth rate (CAGR) of 18%. Specifically, the dermatology segment, encompassing skin tissue engineering, was valued at USD 1.87 billion in 2023 and is expected to grow to USD 7.66 billion by 2030. Similarly, the scar treatment market is forecasted to increase from USD 24 billion in 2022 to USD 64.26 billion by 2032, largely driven by rising aesthetic concerns and the prevalence of scar-inducing injuries. Taken together, these trends underscore the critical need and expanding market potential for advanced skin tissue engineering technologies aimed at enhancing wound healing outcomes and scar management [26]. Despite this growth, commercially available products range from synthetic scaffolds to biologically derived matrices [27,28]; however, many of these products face limitations including limited shelf life, high manufacturing costs, poor biocompatibility, immunogenicity, and insufficient replication of native extracellular matrix (ECM) complexity [28,29,30]. Consequently, there has been a notable shift towards the development and adoption of natural, biomimetic materials that more accurately emulate the skin’s native microenvironment, thus addressing increasing consumer demands for sustainable, biocompatible, and efficacious therapeutic options. In this context, decellularized human dermal ECM has garnered considerable interest as a biomimetic scaffold that closely recapitulates the architecture and biochemical milieu of native skin, thereby supporting more effective tissue regeneration. Moreover, the incorporation of natural biological components into such scaffolds has been increasingly recognized as a critical strategy to augment their functional performance.

In light of these developments, the present study utilizes discarded human skin obtained from surgical procedures, thereby leveraging its origin-to-origin compatibility to minimize immunogenic responses while providing a cost-effective and biologically relevant source for scaffold fabrication. This biological material was subsequently subjected to a decellularization process aimed at eliminating immunogenic cellular components while preserving the native ECM, which is essential for maintaining the scaffold’s bioactivity and structural integrity. In general, decellularization methods are generally classified into chemical, physical, and biological approaches, with detergent-based protocols being most commonly used for skin tissue [15,16,31]. However, it is well-documented that detergents such as SDS and Triton X-100 can compromise collagen integrity and deplete GAGs, leading to weakened mechanical properties and impaired bioactivity of the scaffold [32,33,34,35,36]. For instance, Faulk et al. (2014) reported significant ECM disruption and loss of biomechanical strength with aggressive detergent treatments, underscoring the need for milder yet effective decellularization methods [37]. In light of these challenges, the present study conducted a systematic evaluation of four decellularization protocols for skin tissues. Among them, protocol I utilizes a combination of enzymatic digestion with trypsin, followed by oxidative sterilization using ethanol and hydrogen peroxide, and detergent washes with Triton X-100 combined with EDTA and tris buffer. This approach aims to achieve thorough removal of cellular components while striving to preserve the biochemical and structural integrity of the ECM. Protocol II differs by integrating SDS and isopropanol treatments. SDS is a strong ionic detergent that enhances solubilization of cellular membranes and nuclear materials, while isopropanol contributes to lipid removal and sterilization. Although this protocol may increase decellularization efficiency, it carries a potential risk of disrupting ECM proteins and structure due to the harsher chemical exposure. Protocol III introduces sequential hypotonic and hypertonic treatments combined with detergents such as Triton X-100 and SDS. The hypotonic solution facilitates cell swelling and lysis through osmotic shock, while the hypertonic solution promotes further cellular disruption. The detergents are applied with periodic solution changes to maintain activity. This multi-step process is designed to maximize cellular removal while attempting to minimize damage to ECM components. Protocol IV employs repeated cycles of hypotonic and hypertonic treatments without the use of detergents. This method focuses on mechanical and osmotic cell disruption to maximize decellularization efficacy while minimizing exposure to harsh chemicals. The goal is to better preserve the native biochemical composition and ultrastructure of the ECM, which could be beneficial for scaffold bioactivity. According to our results, Protocol IV, utilizing an osmotic shock-based method involving sequential hypotonic and hypertonic treatments to induce cell lysis without reliance on aggressive detergents or enzymatic agents, being identified as the most effective. Conversely, the lower efficiency of Protocol II is attributed to differences in detergent type and concentration, lack of oxidative and enzymatic treatments, and shorter processing time. Protocol III employs a more comprehensive approach with hypotonic pretreatment and longer detergent exposure, using a combination of Triton X-100 and SDS, resulting in more effective DNA removal compared to Protocol I. Nonetheless, Protocol IV effectively preserves the extracellular matrix’s ultrastructural integrity and biochemical composition by minimizing chemical denaturation and structural disruption, as supported by findings from Ishida et al. (2014) demonstrated that collagen and GAGs were better preserved using a similar osmotic technique [38]. Contrary to this, the degradation of GAGs observed in Protocol III-treated samples is most likely attributable to the extended exposure to ionic detergents, particularly SDS. SDS is known to effectively remove cellular components, but it can also disrupt ECM macromolecules, including GAGs and non-collagenous proteins, due to its strong denaturing properties. In Protocol III, SDS was applied at a relatively high concentration (0.7%) and for a prolonged duration (24 h), with multiple solution refreshes. This aggressive chemical exposure likely led to non-specific solubilization of ECM components, contributing to the significant GAG loss seen in histological staining. In contrast, Protocol IV omits detergent use entirely and instead relies on osmotic stress through hypotonic/hypertonic cycling, which is less damaging to the ECM, thus better preserving GAGs and structural proteins. Additionally, quantitative DNA analysis confirmed that Protocol IV effectively reduced residual DNA content below 30 ng/mg, aligning with established criteria for decellularization adequacy [39]. FTIR further confirmed the biochemical similarity between native and decellularized matrices, reinforcing the notion that Protocol IV maintains key ECM components crucial for cell–matrix interactions and mechanical resilience.

Subsequent to the in-depth characterization of the dECM, this study strategically integrates it into hybrid bio-inks enriched with natural microbiota-derived postbiotics, aiming to develop a more biomimetic and functionally enhanced scaffold through advanced 3D bioprinting techniques. Notably, the successful enzymatic solubilization of dECM into a stable hydrogel structure not only enabled the preservation of native ECM components, which are crucial for facilitating cell adhesion and signaling, but also allowed for extrusion-based 3D printing with remarkably high structural fidelity. Following the fabrication of the hybrid scaffold via 3D bioprinting, its swelling and biodegradation behaviors were systematically analyzed, yielding results of critical importance for scaffold performance in wound healing applications. Specifically, the rapid fluid uptake within the first 3 h and the high swelling ratio of approximately 210% at 72 h indicate excellent hydrophilicity both essential for maintaining a moist wound environment. Such moisture retention facilitates the diffusion of nutrients and oxygen, while simultaneously enhancing cell migration and proliferation, key factors known to accelerate the healing process [3]. This enhanced swelling behavior in G3 is primarily attributed to the incorporation of postbiotic components. Since both G2 and G3 share the same base composition of gelatin and dECM-4 materials rich in hydrophilic functional groups such as hydroxyl, carboxyl, and amino groups, gelatin contributes to water retention through its denatured collagen structure, and dECM-4 provides GAGs and other polar molecules. The notable increase in hydrophilicity in G3 is thus linked to the postbiotic addition, which may contain acidic or polar biomolecules that further promote water absorption, leading to a higher swelling ratio compared to the postbiotic-free G2 formulation. These findings are consistent with previous reports demonstrating that high swelling ratios in hydrogel-based scaffolds effectively mimic the fluid-exchange properties of native dermal tissues, thereby promoting favorable cellular responses [40]. Moreover, the scaffold’s-controlled degradation rate, demonstrated by a 43% mass loss over 7 days, further supports its suitability for dynamic tissue environments, where gradual scaffold resorption is necessary to allow for new tissue ingrowth. This degradation profile aligns well with the physiological timeline of wound healing and mirrors trends reported in natural polymer-based scaffolds including those incorporating alginate, gelatin, or ECM-derived components [41]. However, the incorporation of both dECM and postbiotics into the fabricated scaffolds led to a slight reduction in tensile and compressive strength, a finding consistent with well-documented reports that frequently observe this trade-off in dECM-based hydrogels [42].

Importantly, the integration of postbiotics conferred robust antibacterial activity against clinically relevant pathogens, including MRSA and *Pseudomonas aeruginosa*, which are commonly implicated in chronic wound infections. This finding aligns well with recent evidence highlighting the antimicrobial efficacy of postbiotics, particularly those derived from *Lactobacillus* species in wound healing process [43,44,45]. Moreover, it offers a distinct advantage over previous studies that utilized live probiotics, which have raised safety concerns, particularly among immunocompromised patient populations [46]. Beyond their antimicrobial properties, postbiotic-loaded dECM scaffolds effectively support keratinocyte proliferation and viability, which are essential for faster re-epithelialization during wound healing. Over a 7-day biocompatibility assessment, no cytotoxicity was observed; rather, the scaffold promoted robust cell growth and survival, confirming its suitability as a cellular support matrix. This is largely attributed to the sustained bioavailability of postbiotics within the scaffold, allowing continuous release of bioactive molecules that enhance keratinocyte function and viability which are key drivers of epidermal regeneration [13,47].

To the best of our knowledge, this study represents the first comprehensive comparison of four distinct decellularization protocols specifically tailored for human skin tissue, offering a broad and systematic perspective that has been largely overlooked in previous research. This protocol-based evaluation is of particular significance, as the identified optimal method (Protocol IV) not only ensured substantial DNA removal but also achieved superior preservation of essential ECM components which are critical for maintaining the biological functionality of the scaffold. Therefore, this methodological framework provides a robust foundation for future tissue engineering strategies and has the potential to guide subsequent research in the development of ECM-based biomaterials. Building upon this optimized dECM, we further advanced the field by incorporating postbiotics into a 3D-bioprinted scaffold, marking a novel and innovative approach in chronic wound management. Although the beneficial effects of postbiotics have been well documented in conventional topical formulations such as creams and gels [12,48,49], their application within bio-fabricated constructs particularly in the context of 3D bioprinting has remained virtually unexplored. By introducing postbiotics into this newly developed scaffold system, our study bridges this gap and expands the potential therapeutic utility of postbiotics beyond surface applications. In this context, our findings underscore the synergistic advantages of combining dECM with postbiotics within a 3D-bioprinted platform, thereby addressing both the regenerative and antimicrobial requirements of chronic wound healing. Although the in vitro outcomes are highly promising, comprehensive in vivo studies are essential to validate the therapeutic efficacy, biocompatibility, and long-term safety of the postbiotic-enriched scaffolds in clinically relevant settings. Ultimately, this work lays important groundwork for the development of next-generation, multifunctional scaffolds tailored for complex, non-healing wounds.

## Figures and Tables

**Table 1:** Protocols used in human skin decellularization.

Protocol I	Protocol II	Protocol III	Protocol IV
Wash with 1× PBS for 1 h	Wash with distilled water (3×)	Wash with distilled water (3×)	Wash with 1× PBS for 1 h
0.25% Trypsin-EDTA for 6 h	0.25% Trypsin-EDTA for 6 h	Hypotonic solution (10 mM Tris-HCl, 5 mM EDTA, 1 µM PMSF) for 12 h	Hypotonic buffer (10 mM Tris-HCl, 5 mM EDTA, 1 µM PMSF) for 8 h
Water rinse (3×)	Water rinse (3×)	Hypertonic solution (0.5% Triton X-100) for 24 h	Hypertonic buffer (50 mM Tris-HCl, 1 M NaCl, 10 mM EDTA, 1 µM PMSF) for 8 h
70% ethanol for 10 h	0.1% SDS in 70% isopropanol for 6 h	Rinse with 1× PBS for 2 h	Repeat steps 2 and 3
3% H_2_O_2_ for 15 min	Water rinse (5×)	0.7% SDS for 24 h, refresh every 6 h	Wash 3× with 1× PBS (2 h each)
Water rinse (3×)	1% Triton X-100 in 70% isopropanol for 12 h	0.05% peracetic acid and 3% ethanol for 3 h	
1% Triton X-100 + 0.26% EDTA + 0.69% Tris for 22 h	Water rinse (5×)	Wash with 1× PBS	
Water rinse (3×)	100% isopropanol for 12 h		
0.1% peracetic acid + 4% ethanol for 2 h	Water rinse (6×)		
Rinse with PBS (2×) and dH_2_O (2×)			

## Data Availability

The datasets of 16S rRNA gene sequencing generated during the recent study have been deposited in the NCBI GenBank repository under the accession numbers PP951925 (*Limosilactobacillus reuteri* EIR/Spx-2). Other data supporting the findings of this study are available from the corresponding author upon reasonable request.
